# Evidence of plasma biomarkers indicating high risk of dementia in cognitively normal subjects

**DOI:** 10.1038/s41598-022-05177-z

**Published:** 2022-01-24

**Authors:** Ming-Chyi Pai, Chau-Chung Wu, Yi-Chou Hou, Jiann-Shing Jeng, Sung-Chun Tang, Wei-Che Lin, Cheng-Hsien Lu, Ming-Jang Chiu, Ta-Fu Chen, Sui-Hing Yan, Chaur-Jong Hu, Shieh-Yueh Yang

**Affiliations:** 1 Division of Behavioral Neurology, Department of Neurology, National Cheng Kung University Hospital, College of Medicine, National Cheng Kung University, Tainan, 701 Taiwan; 2 Division of Cardiology, Department of Internal Medicine, National Taiwan University Hospital, Taipei, 100 Taiwan; 3 Department & Graduate Institute of Medical Education & Bioethics, College of Medicine, National Taiwan University, Taipei, 100 Taiwan; 4 Graduate Institute of Clinical Medicine, College of Medicine, Taipei Medical University, Taipei, 110 Taiwan; 5 Department of Internal Medicine, Cardinal Tien Hospital, New Taipei City, 231 Taiwan; 6 School of Medicine, Fu Jen Catholic University, New Taipei City, 242 Taiwan; 7 Department of Neurology, National Taiwan University Hospital, Taipei, 100 Taiwan; 8 Department of Diagnostic Radiology, Kaohsiung Chang Gung Memorial Hospital, Chang Gung University College of Medicine, Kaohsiung, 833 Taiwan; 9 Department of Neurology, Kaohsiung Chang Gung Memorial Hospital, Chang Gung University College of Medicine, Kaohsiung, 833 Taiwan; 10 Graduate Institute of Brain and Mind Sciences, College of Medicine, National Taiwan University, Taipei, 100 Taiwan; 11 Department of Psychology, National Taiwan University, Taipei, 106 Taiwan; 12 Graduate Institute of Biomedical Electronics and Bioinformatics, National Taiwan University, Taipei, 106 Taiwan; 13 Department of Neurology, Far Eastern Memorial Hospital, New Taipei City, 220 Taiwan; 14 Department of Neurology, School of Medicine, College of Medicine, Taipei Medical University, Taipei, 106 Taiwan; 15 Department of Neurology, Dementia Center, Shuang Ho Hospital, Taipei Medical University, New Taipei City, 235 Taiwan; 16 Graduate Institute of Neural Regenerative Medicine, College of Medical Science and Technology, Taipei Medical University, Taipei, 106 Taiwan; 17 Taipei Neuroscience Institute, Taipei Medical University, Taipei, 106 Taiwan; 18 MagQu Co., Ltd., New Taipei City, 231 Taiwan

**Keywords:** Neuroscience, Biomarkers, Diseases, Neurology, Risk factors

## Abstract

Subjects with comorbidities are at risk for neurodegeneration. There is a lack of a direct relationship between comorbidities and neurodegeneration. In this study, immunomagnetic reduction (IMR) assays were utilized to assay plasma Aβ_1–42_ and total tau protein (T-Tau) levels in poststroke (PS, n = 27), family history of Alzheimer’s disease (ADFH, n = 35), diabetes (n = 21), end-stage renal disease (ESRD, n = 41), obstructive sleep apnea (OSA, n = 20), Alzheimer’s disease (AD, n = 65). Thirty-seven healthy controls (HCs) were enrolled. The measured concentrations of plasma Aβ_1–42_ were 14.26 ± 1.42, 15.43 ± 1.76, 15.52 ± 1.60, 16.15 ± 1.05, 16.52 ± 0.59, 15.97 ± 0.54 and 20.06 ± 3.09 pg/mL in HC, PS, ADFH, diabetes, ESRD, OSA and AD groups, respectively. The corresponding concentrations of plasma T-Tau were 15.13 ± 3.62, 19.29 ± 8.01, 17.93 ± 6.26, 19.74 ± 2.92, 21.54 ± 2.72, 20.17 ± 2.77 and 41.24 ± 14.64 pg/mL. The plasma levels of Aβ_1–42_ and T-Tau in were significantly higher in the PS, ADFH, diabetes, ESRD and OSA groups than controls (Aβ_1–42_ in PS: 15.43 ± 1.76 pg/mL vs. 14.26 ± 1.42 pg/mL, p < 0.005; T-Tau in PS: 19.29 ± 8.01 vs. 15.13 ± 3.62 pg/mL, p < 0.005, Aβ_1–42_ in ADFH: 15.52 ± 1.60 pg/mL vs. 14.26 ± 1.42 pg/mL, p < 0.001; T-Tau in ADFH: 17.93 ± 6.26 vs. 15.13 ± 3.62 pg/mL, p < 0.005, Aβ_1–42_ in diabetes: 16.15 ± 1.05 pg/mL vs. 14.26 ± 1.42 pg/mL, p < 0.001; T-Tau in diabetes: 19.74 ± 2.92 vs. 15.13 ± 3.62 pg/mL, p < 0.001, Aβ_1–42_ in ESRD: 16.52 ± 0.59 pg/mL vs. 14.26 ± 1.42 pg/mL, p < 0.001; T-Tau in ESRD: 21.54 ± 2.72 vs. 15.13 ± 3.62 pg/mL, p < 0.001, Aβ_1–42_ in OSA: 15.97 ± 0.54 pg/mL vs. 14.26 ± 1.42 pg/mL, p < 0.001; T-Tau in OSA: 20.17 ± 2.77 vs. 15.13 ± 3.62 pg/mL, p < 0.001). This evidence indicates the high risk for dementia in these groups from the perspective of plasma biomarkers.

## Introduction

In 2020, the global prevalence of dementia was approximately 8%, i.e., 30 million patients^1^. Alzheimer’s disease (AD) is the most common dementia. With the aging of society, the number of dementia patients is expected to be more than 100 million in 2050^2–4^. Management and health care for dementia are definitely a large economic burden at present. This burden will become more serious in the future if there is no action taken to cure or prevent dementia. Unfortunately, no curative treatment is currently available for dementia. Several pharmaceutical companies continue to develop new drugs for the treatment of dementia. On the other hand, prevention efforts for dementia attract the attention of medical doctors or researchers. Exciting results have been reported to demonstrate the feasibility of delaying the onset of dementia using aerobic exercise, nutrition adjustments, or sleeping quality improvement^5–8^. The delay could effectively reduce the population of dementia in the future.

People with risk factors are strongly suggested to take such preventive actions when they are still cognitively normal. These people are referred to as high risk for dementia (HRD). Most of these risk factors are similar to those for cardiovascular disease, such as hypertension, hyperlipidemia, diabetes, and obesity^9–13^. According to published papers, people with these risk factors at midlife have relatively higher possibilities of suffering from dementia when aged more than 65 years^9–13^. In addition to increasing cardiovascular risk, family history, hypoxia, and other comorbidities are potential risk factors for neurodegeneration^14–16^. Some models using scores of these risk factors at midlife to predict the occurrence of dementia with aging have been developed for clinical assessments^9,17^. However, there is a lack of a direct relationship between these risk factors and dementia pathology.

Amyloid positron emission tomography (PET) is the most direct assessment of dementia pathology, especially AD^18–20^. Due to its low availability and high cost, amyloid PET examinations in HRD are difficult to perform. Significant correlations between biomarkers in cerebrospinal fluid (CSF) and amyloid PET results have been demonstrated^21–23^. CSF biomarker assays are an alternative way to explore dementia pathology in HRD. Lumbar puncture for sampling CSF, however, has side effects, which limit the utility of CSF biomarkers in clinical practice. Recently, assays for dementia biomarkers in plasma have become available^24–29^. Significant correlations between plasma amyloid β (Aβ) levels and amyloid PET results have been reported^30–32^. This implies that dementia pathology in HRD could be explored using plasma biomarker assays. In this study, immunomagnetic reduction (IMR) assays were utilized to assess plasma Aβ_1–42_ and total Tau protein (T-Tau) levels in HRD. The enrolled HRD included poststroke (PS) subjects, individuals with a family history of AD (ADFH), and patients with diabetes, end-stage renal disease (ESRD) and obstructive sleep apnea (OSA).

## Methods

### Recruitment of subjects

In this study, there were 246 subjects enrolled at six hospitals in Taiwan, and all studies were approved by the hospital institutional review boards. Thirty-seven heathy controls (HCs) were enrolled. There was no hypertension, hyperlipidemia, diabetes, obesity, cardiovascular disease, metabolic disease, brain ischemia, or renal disease in any of the HCs. There were 27 PS patients with normal cognition^33^. Thirty-five cognitively normal people had a first-degree family history of AD. Twenty-one subjects had diabetes mellitus (diabetes). Forty-one patients had end-stage renal disease (ESRD)^34^. Twenty subjects suffered from obstructive sleep apnea (OSA). Sixty-five patients with AD were enrolled^35^. The AD patients were diagnosed according to NIA-AA 2011 guidelines. The institutional review boards of all the joining hospitals approved this study. All participants provided written informed consent prior to study enrollment.

### Plasma preparation

A 6-mL EDTA blood collection tube was used for blood draw, followed by centrifugation of the collected blood at a speed ranging from 1500 to 2500 g for 15 min at room temperature with the aid of a swing-bucket rotor. From each EDTA tube, 1 mL of plasma was transferred to 0.5-mL microcentrifuge tubes and stored at − 80 °C until biomarker assays were performed. Plasma was frozen no later than 4.5 h after blood draw. All plasma samples were shipped in a dry-ice package to MagQu Co., Ltd. in New Taipei City, Taiwan, for the blind assessment of plasma biomarkers.

### Plasma biomarker assays

Plasma biomarkers were assayed with immunomagnetic reduction (IMR). IMR kits (MF-AB2-0060, MF-TAU-0060, MagQu) and the IMR analyzer (XacPro-S, MagQu) were used for assaying Aβ_1–42_ and T-Tau. For assaying Aβ_1–42_/T-Tau, 60/80 μL reagent was mixed with 60/40 μL plasma for each measurement. Duplicated measurements were conducted for each biomarker per sample. The mean value of the duplicated measurements was reported for each biomarker for a subject. The mean, repeatability coefficient of variation (%CV), within-lab %CV and lowest limit of quantification (LLOQ) of Aβ_1–42_ and T-Tau IMR assay are shown in Table 1.

**Table 1:** The mean, repeatability coefficient of variation (%CV), within-lab %CV and lowest limit of quantification (LLOQ) of Aβ_1–42_ and T-Tau IMR assay.

Item tested	Mean of measured target sprotein concentrations (pg/mL)	Standard deviation (%CV)	
		Repeatability	Within-Lab
Aβ_1–42_ pool 1	100.82	1.87 (1.9)	6.07 (6.0)
Aβ_1–42_ pool 2	10.20	0.65 (6.4)	0.68 (6.7)
Tau pool 1	101.44	1.71 (1.7)	4.08 (4.0)
Tau pool 2	10.25	0.57 (5.6)	0.56 (5.5)

### Statistical methods

Continuous variables for each measurement are presented as the mean ± standard deviation. Continuous variables were compared using t-tests, and the *p* values were determined.

### Ethical standards

The study was started and conducted after approval of the study protocol by the regional ethical committee in all the joined hospitals, including National Cheng Kung University Hospital, National Taiwan University Hospital, Cardinal Tien Hospital, Kaohsiung Chang Gung Memorial Hospital, Taipei City Hospital, and Shuang Ho Hospital, approved the study protocol, and the study was carried out in accordance with relevant guidelines and regulations, including the World Medical Association (WMA) Declaration of Helsinki. Since the patients could not themselves consent to the study, a written informed consent was obtained from the patient’s closest relative.

## Results

The subjects with diabetes had an AC sugar level of 127.6 ± 26.7 g/dL and HbA1c level of 6.51 ± 0.52%. The individuals with OSA had an apnea–hypopnea index (AHI) of 54.6 ± 17.5.

Demographic information of the enrolled subjects is listed in Table 1. The ages of the HC, PS, ADFH, diabetes, ESRD, OSA and AD groups were 63.1 ± 12.4, 70.7 ± 6.9, 59.75 ± 4.7, 75.8 ± 7.3, 62.5 ± 7.8, 40.3 ± 8.7 and 78.8 ± 7.3 years, respectively. Except for ADFH and ESRD, all other groups had significant differences in age compared to the HC. Although the ages among groups were not matched, this was not a crucial point in exploring the differences in plasma biomarker levels among groups because the plasma Aβ_1–42_ and T-Tau levels measured with immunomagnetic reduction were not significantly age dependent^36^.

The subjects in the HC, PS, ADFH, ESRD and OSA groups were cognitively normal, had a clinical dementia ranking (CDR) of zero and a Mini-Mental State Examination (MMSE) score higher than 26. The CDR of every subject with AD was equal to or higher than 0.5 (1.11 ± 0.63). The MMSE score of the AD patients was 18.7 ± 4.1. The AD group clearly showed cognitive impairment.

**Figure 1:**
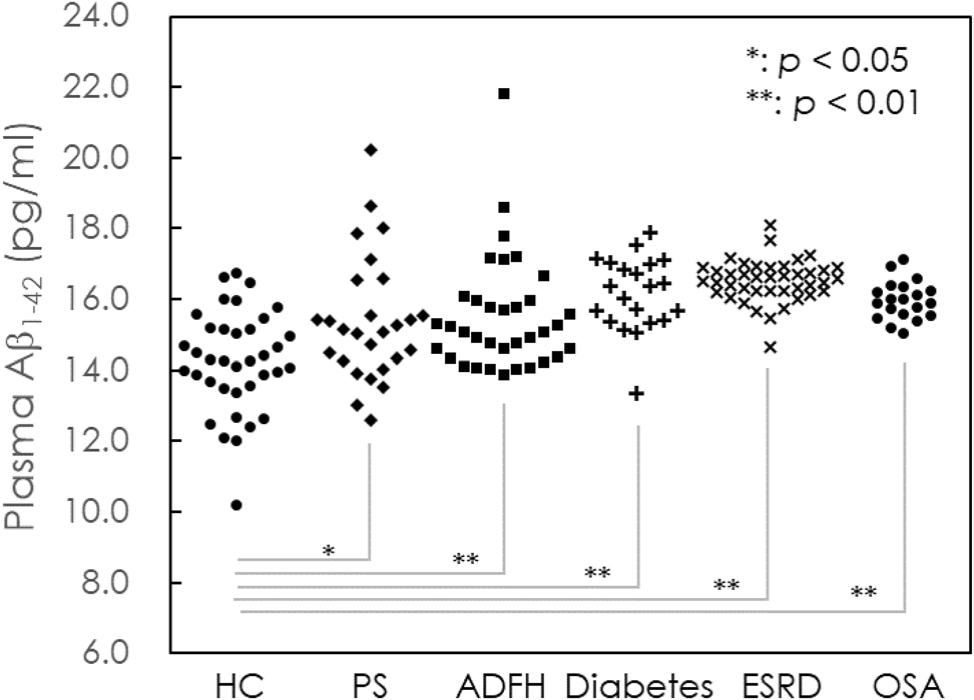
Dot plot of measured levels of Aβ_1–42_ in the plasma of healthy control (HC), poststroke subjects (PS), individuals with a family history of Alzheimer’s disease (ADFH), and patients with end-stage renal disease (ESRD), obstructive sleep apnea (OSA) and Alzheimer’s disease (AD). *p < 0.05 and **p < 0.01 versus healthy control.

The measured concentrations of plasma Aβ_1–42_ were 14.26 ± 1.42 pg/mL in HC, 15.43 ± 1.76 pg/mL in PS, 15.52 ± 1.60 pg/mL in ADFH, 19.74 ± 2.92 pg/mL in diabetes, 16.52 ± 0.59 pg/mL in ESRD, 15.97 ± 0.54 pg/mL in OSA and 20.06 ± 3.09 pg/mL in AD groups. Notably, the HC group showed relatively low levels of plasma Aβ_1–42_ (Fig. 1). The levels gradually increase across groups from HC to PS to ADFH to OSA to ESRD and to AD. Through t-test analyses using Aβ_1–42_ levels in HCs as a reference, the PS group showed a *p* value lower than 0.05, while the other groups showed *p* values lower than 0.001. This implied that groups, such as the PS, ADFH, ESRD and OSA groups, had significantly higher concentrations of plasma Aβ_1–42_ than the HC group but lower concentrations than the AD group of patients. According to the results in published papers, elevations in plasma Aβ_1–42_ levels were shown to be associated with amyloid pathology in the brain^30,31,37,38^. Thus, PS, ADFH, ESRD and OSA are high-risk factors for the accumulation of amyloid in the brain. Unfortunately, amyloid PET imaging was not available to explore amyloid deposition in the brain for the subjects in the PS, ADFH, ESRD and OSA groups in this study.

Another typical biomarker for dementia, especially for AD, is tau protein. The levels of plasma total tau protein (T-Tau) were determined for all subjects. As listed in Table 2, the T-Tau level in the HC group was 15.13 ± 3.62 pg/mL; PS, 19.29 ± 8.01 pg/mL; ADFH, 17.93 ± 6.26 pg/mL; diabetes 19.74 ± 2.92 pg/mL; ESRD, 21.54 ± 2.72 pg/mL; OSA, 21.54 ± 2.72 pg/mL; and AD, 41.24 ± 14.64 pg/mL. The results showed that all PS (*p* < 0.05), ADFH (*p* < 0.001), diabetes (*p* < 0.001), ESRD (*p* < 0.001) and OSA (*p* < 0.001) patients had significantly higher levels of plasma T-Tau than HCs but significantly lower levels than AD patients. Hence, from the perspective of plasma biomarkers, these groups are at high risk for neurodegeneration.

**Table 2:** Numbers, age, and measured plasma biomarker levels of enrolled subjects.

Group	N (female)	Age (years)	Aβ_1–42_ (pg/mL)	T-Tau (pg/mL)	Aβ_1–42_xT-Tau	MMSE	CDR
HC	37 (23)	63.1 ± 12.4	14.26 ± 1.42	15.13 ± 3.62	216.78 ± 60.89	> 26	0
PS	27 (11)	70.7 ± 6.9**	15.43 ± 1.76*	19.29 ± 8.01*	304.06 ± 150.66*		
ADFH	35 (25)	59.75 ± 4.7	15.52 ± 1.60**	17.93 ± 6.26**	285.88 ± 133.32*		
Diabetes	21 (8)	75.8 ± 7.3***	16.15 ± 1.05**	19.74 ± 2.92**	320.52 ± 61.18**		
ESRD	41 (16)	62.5 ± 7.8	16.52 ± 0.59**	21.54 ± 2.72**	356.59 ± 52.82**		
OSA	20 (20)	40.3 ± 8.7***	15.97 ± 0.54**	20.17 ± 2.77**	322.31 ± 47.15**		
AD	65 (35)	78.8 ± 8.8***	20.06 ± 3.09**	41.24 ± 14.64**	824.34 ± 302.2**	18.7 ± 4.1	1.11 ± 0.63

*HC* healthy control, *PS* poststroke, *ADFH* family history of Alzheimer’s disease, *ESRD* end-stage renal disease, *OSA* obstructive sleep apnea, *AD* Alzheimer’s disease, *Aβ* amyloid β, *T-Tau* total tau protein.

*p < 0.05 with respect to HC; **p < 0.01 with respect to HC; ***p < 0.001 with respect to HC.

## Discussion

As shown in Table 1, the PS, ADFH, diabetes, ESRD, and OSA groups had higher levels of plasma Aβ_1–42_ and T-Tau than the HC group but lower levels than the AD group. This implied that there is a high suspicion of individuals in these groups progressing to dementia. Other previous analytic platforms show lower Aβ_1–42_ concentrations in AD compared with other diagnostic groups^39,40^. However, the different results may result from differences in detection principles and target proteins. Most of detection systems mainly recognizes Aβ42 monomer and a small amount of oligomer, but because it requires paired antibodies, the signal of oligomers may be underestimated. However, immunomagnetic reduction (IMR) uses only a single antibody and could identify both Aβ42 monomers and oligomers, allowing more target proteins to be captured. Several countries, including Sweden, Taiwan, United Kingdom and the United States, have used IMR to obtain similar data^41–43^. The above data prove that the results are reproducible and reproducible. According to studies by other labs, increased plasma Aβ oligomers level was measured in AD patients^37,44^. These results are similar to the IMR measurement results, so IMR may be mainly recognized to Aβ42 oligomer. The recognized Aβ categories still need to be further studied.

A follow-up study is needed to investigate the occurrence of dementia in these groups. It would be better to simultaneously observe the changes in plasma biomarker levels in the context of a longitudinal study. In fact, several papers have revealed the relatively high occurrence of dementia in PS individuals or those with ADFH, diabetes, ESRD, or OSA. For example, Pendlebury et al. reported that PS dementia occurs in 7% of PS patients 1 year after a stroke^45^. The incidence of cognitive impairment was 10–40% among those with ESRD^46^. As reported by Tufik et al., OSA has been associated with several comorbidities, including cognitive impairment^47^. A previous study showed that OSA patients exhibited significantly higher serum Aβ_1–42_ levels than age- and sex-matched controls^48^. Scarabino et al. showed that a family history of dementia was associated with an increased risk of AD, with an odds ratio of 2.71 (*p* < 0.05)^15^. Furthermore, an individual having a first-degree relative with dementia was significantly associated with risk of AD (odds ratio = 2.9, *p* < 0.05). Although the associations between these diseases and dementia have been discussed, the results of plasma Aβ_1–42_ and T-Tau levels reported in this study demonstrate the high risk for dementia with biological evidence.

Plasma biomarkers are promising for assessing the risk of AD. The values of Aβ_1–42_xT-Tau in plasma have been validated as a clinical index for assessing AD^35,42,49^. Several research groups announced that the cutoff values of plasma Aβ_1–42_xT-Tau levels for discriminating amnesic mild cognitive impairment and AD patients from cognitively normal controls range from 382 to 455 pg^2^/mL^2^^35,42,49^. The difference in the cutoff levels among reports might be due to some possible causes such as AD severity of enrolled subjects, subjective assessments on neuropsychological tests, plasma preparation, etc. In this study, the subjects showing plasma Aβ_1–42_xT-Tau levels higher than 382 pg^2^/mL^2^ were referred to subhealth.

**Figure 2:**
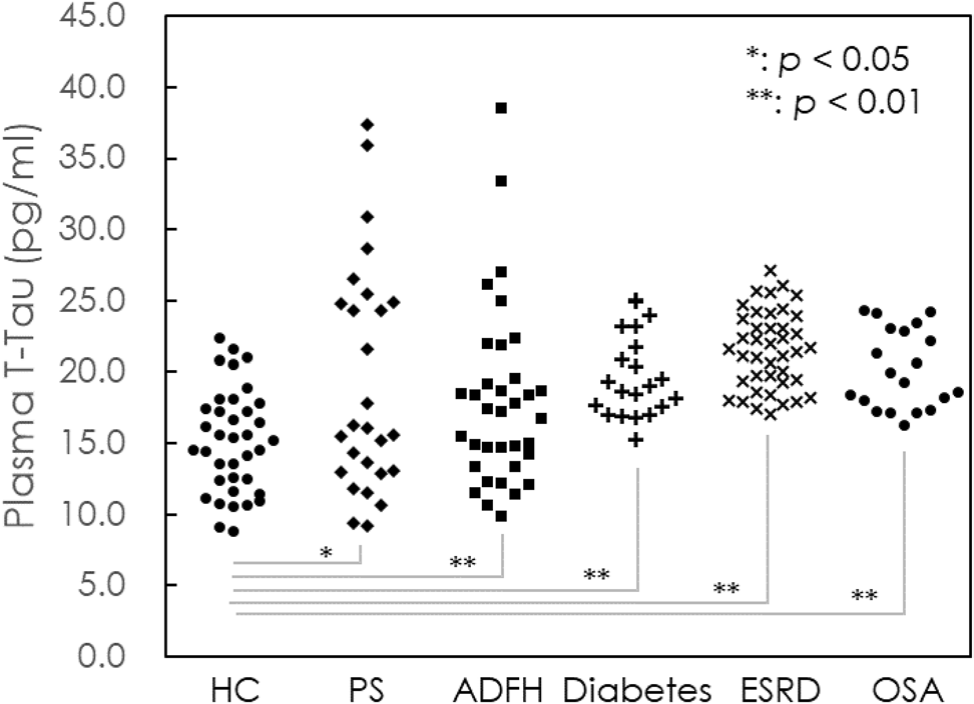
Dot plot of measured levels of T-Tau in the plasma of HCs, PS, ADFH, and ESRD, OSA and AD.

**Figure 3:**
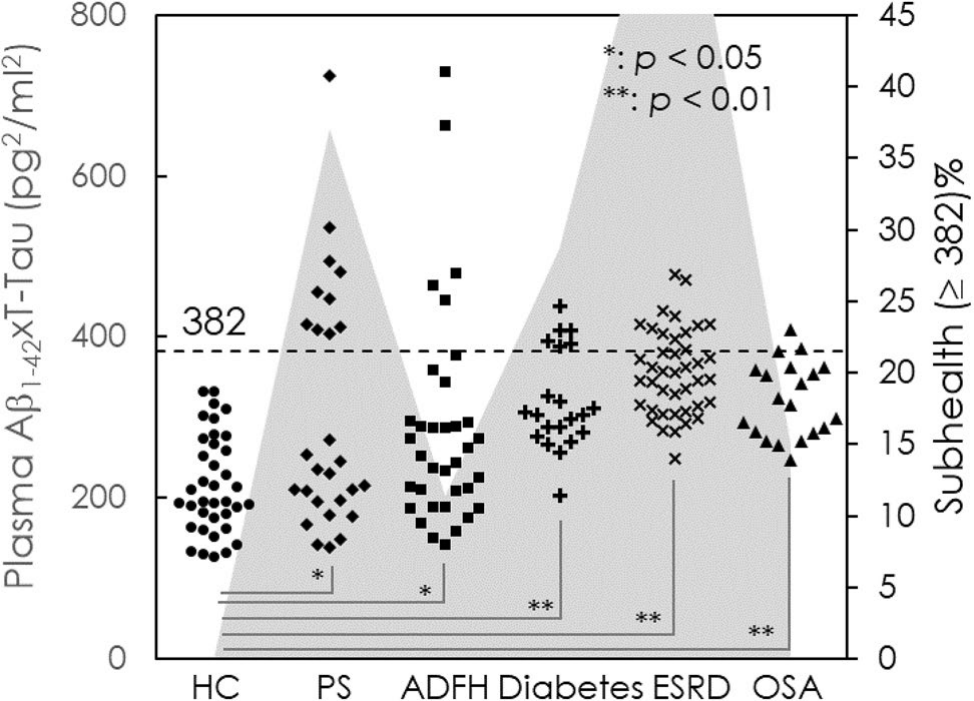
Dot plot of measured levels of Aβ_1–42_xT-Tau in the plasma of healthy controls (HCs), poststroke subjects (PS), individuals with a family history of Alzheimer’s disease (ADFH), and patients with end-stage renal disease (ESRD), obstructive sleep apnea (OSA) and Alzheimer’s disease (AD). The prevalence of subhealth (Aβ_1–42_xT-Tau ≥ 382) in each group is plotted with a gray background.

The concentrations of Aβ_1–42_ in plasma for individuals in different high-risk group using IMR are shown in Fig. 1. The concentrations of Aβ_1–42_ in the high-risk group were lower than that in AD group (20.06 ± 3.09 pg/mL), but higher than that in HC group (14.26 ± 1.42 pg/mL). This indicates that the microenvironment of the brain has changed compared to normal individuals, resulting in an increase in the amount of Aβ_1–42._ The concentrations of plasma Tau for individuals in high-risk group are shown in Fig. 2. The concentrations of Tau in the high-risk group were lower than that in AD group (15.13 ± 3.62 pg/mL), but higher than that in HC group (41.24 ± 14.64 pg/mL). This suggests that the high-risk group has some neurological damage compared to the HC group, leading to an increase in the amount of Tau expression. The dot plot of plasma Aβ_1–42_xT-Tau for individuals in each group is shown in Fig. 3. The value of 382 pg^2^/mL^2^ is indicated with the dashed line. There were no individuals with plasma Aβ_1–42_xT-Tau levels higher than 382 among the HCs. However, some proportion of subjects in the PS, ADFH, diabetes, ESRD and OSA groups showed Aβ_1–42_xT-Tau levels higher than 382 pg^2^/mL^2^. The prevalence of subhealth in each group was analyzed and plotted with the gray background in Fig. 3. There were 37% of PS subjects and 11.4% of ADFH subjects considered subhealth. In the diabetes and ESRD groups, the prevalence of subhealth was 28.6% and 29.3%, respectively. The subhealth occurrence in the OSA group was found to be 15%. Based on the results in Fig. 1, PS is the riskiest factors for dementia. The fact that 37% of PS subjects were classified as subhealth may explain why the occurrence of poststroke dementia is so high^45^. Notably, ADFH showed a lower prevalence of subhealth than OS. Moreover, the ADFH group had only one-third of the subhealth prevalence observed in the PS group and half the prevalence observed in the diabetes and ESRD groups. This might imply that biological causes such as hypertension, hypoxia or electrolyte imbalance frequently found in PS, diabetes, ESRD and OSA patients are more dominant in relation to the risk for dementia than family history.

### Limitation

The total numbers of enrolled subjects in each group are relatively limited. More subjects should be enrolled for validating the percentages of high-risk dementia in each group.

## Conclusion

The plasma levels of Aβ_1–42_ and T-Tau in the PS, ADFH, diabetes, ESRD, and OSA groups were relatively high compared to those in the HC group but were lower than those in the AD group. On average, the prevalence of subhealth in these groups was 16.7%. This evidence indicated the high risk for dementia in these groups from the perspective of plasma biomarkers.

## Data Availability

The dataset generated and analyzed in the current study is available from the corresponding author on reasonable request.
